# Nervous Diseases and Lunacy Law

**Published:** 1901-11-16

**Authors:** 


### The Hospital

A Journal of

The Medical Sciences and Hospital Administration.

Vol. XXXI.—No. 790.] Edited by Sir Henry Burdett, K.C.B., and Solomon C. Smith, M.D., M.R.C.P. [November 16, 1901.

### Nervous Diseases and Lunacy Law.

The more we see of the working of the Lunacy Acts the more impatient we become at the legal restrictions which compel us to make a rigid separation between diseases of the mind and those of all the rest of the nervous system. The whole position of affairs is on the very face of it absurd. We have to do with a wonderful machine built up of tissues and of cells, each with its own semi-independent vitality, yet all subordinated to the good of the whole by being linked together by the network of the nervous system, which through infinite gradations of complexity passes from the mere co-ordination of organs to the performance of mental functions and the regulation of conduct. Working upwards from the simple to the more complex, physiologists and physicians labour to elucidate the phenomena and to cure the diseases of the nervous system, and so long as these diseases only affect the action of muscles, the function of glands, the general well-being of the economy, and even when, ascending to the higher centres, they abolish the faculty of mind, the physician has free play, and, unfettered by law, is allowed to do his best. Directly, however, disease produces delusions or touches conduct the psychologist and the alienist come upon the field, and instead of extending their inquiries still higher along the line of the nervous system, turn right about, take hold of the stick at the other end, and, starting at the "mind," work downwards through that long series of mental disorders which are included under the term insanity, every step in connection with which is strictly regulated by law. In the dead-house the physician and the alienist meet over the general paralytic and discuss the morbid changes in the common language of pathology, but in almost all else they are on opposite sides of the hedge—a useless hedge, an obstructive hedge, and one which is maintained by laws passed when madhouses were a scandal and the humanising influence of science had hardly commenced to penetrate their walls. Not only is the effect of all this disastrous in regard to the care and treatment of those who are doubtfully insane, but it is to be feared that the harshness and rigidity of the law not infrequently lead to its provisions being so circumvented in regard to those who are really insane that its very object is defeated.

Speaking recently on the subject of the "single care" of private patients, Dr. T. Seymour Tuke,[1] who certainly may be said to speak with knowledge, said that there could be no doubt that there are many people maintained in all kinds of places "unknown to the Commissioners"—unknown therefore to the law—unregistered in any way, and under the charge of persons whose qualifications for undertaking this work are of the smallest. But there is another phase of the same question. There is a tendency nowadays to gloss over what is in reality actual insanity by calling it by euphemistic epithets. The words "borderland," "hystero-mania," and "neurasthenia," are stretched to such an extent that the self-same treatment which one man may use in a licensed house, under strict supervision and with a really legal responsibility, may be and is used by others, if they choose to take the risk, absolutely free from all restriction and supervision, with no "casebook" to keep, no special reports to make, and no account to give of the remuneration that they receive. People like to be told that the case of their friend or relative is simply one of nervous disorder, "and under this idea," Dr. Tuke says, "they consent to allow what is virtually restraint, which cannot be called legal, to be applied."

Of late years there has been a great increase in the number of medical men who, forced by the persuasion of those who have the dread of that terrible word "certification" before them, treat all kinds and varieties of cases which fall under the head of nervous disease. Many of these, being honourable men, try to keep the balance between, and to observe legal figment which separates, serious nervous disorder and actual insanity. "But," says Dr. Tuke, "there are others; and when one hears of this great array of nursing homes, rest houses, nerve homes, and the other establishments which openly exist for the purpose of someone making a livelihood out of the care of their inmates, when one hears that it is allowable to take patients and forbid them to leave their beds for months together, to keep their relatives away from them, and to cause them to submit themselves to the sway of nurses and companions, one cannot help asking oneself sometimes where the liberty of the subject is considered, and what real difference there is between this and actual asylum treatment, except that in the one case the patient is not known to, or visited by the proper authorities, and in the other he is." Here we have forcibly depicted one of the great evils of the present condition of the Lunacy Law. Not all these people whom Dr. Tuke speaks of are insane, many of them are on the borderland, a land which cannot be defined. The law, however, demands that it shall be defined, and therefore the law is broken.

[1] Brit. Med. Journ., Oct. 26.

